# Factors affecting in-hospital mortality of non-tuberculous mycobacterial pulmonary disease

**DOI:** 10.1186/s12879-021-06395-y

**Published:** 2021-07-20

**Authors:** Goh Tanaka, Taisuke Jo, Hiroyuki Tamiya, Yukiyo Sakamoto, Wakae Hasegawa, Hiroki Matsui, Kiyohide Fushimi, Hideo Yasunaga, Takahide Nagase

**Affiliations:** 1 Department of Respiratory Medicine, The University of Tokyo, 7-3-1 Hongo, Bunkyo-ku, Tokyo, 113-8655 Japan; 2 Department of Health Services Research, Graduate School of Medicine, The University of Tokyo, Tokyo, Japan; 3 Department of Clinical Epidemiology and Health Economics, School of Public Health, Graduate School of Medicine, The University of Tokyo, Tokyo, Japan; 4 Department of Health Policy and Informatics, Tokyo Medical and Dental University Graduate School of Medicine, Tokyo, Japan

**Keywords:** Non-tuberculous mycobacterial disease, Hospital mortality, Body mass index

## Abstract

**Background:**

The incidence and prevalence of non-tuberculous mycobacterial pulmonary disease (NTM-PD) are reportedly increasing in many parts of the world. However, there are few published data on NTM-PD-related death. Using data from a national inpatient database in Japan, we aimed in this study to identify the characteristics of patients with NTM-PD and clinical deterioration and to identify risk factors for in-hospital mortality.

**Methods:**

We examined data from the Diagnosis Procedure Combination (DPC) database in Japan from July 2010 to March 2014. We extracted data for HIV-negative NTM-PD patients who required unscheduled hospitalization. We evaluated these patients’ characteristics and performed multivariable logistic regression analysis to identify risk factors for all-cause in-hospital mortality.

**Results:**

A total of 16,192 patients (median age: 78 years; women: 61.2%) were identified. The median body mass index (BMI) was 17.5 kg/m^2^ (IQR 15.4–20.0). All cause In-hospital death occurred in 3166 patients (19.6%). The median BMI of the patients who had died was 16.0 kg/m^2^ (IQR 14.2–18.4). Multivariable analysis revealed that increased mortality was associated with male sex, lower BMI, lower activities of daily living scores on the Barthel index, hemoptysis, and comorbidities, including pulmonary infection other than NTM, interstitial lung disease, pneumothorax, and malignant disease.

**Conclusions:**

We found associations between being underweight and having several comorbidities and increased in-hospital mortality in patients with NTM-PD. Preventing weight loss and management of comorbidities may have a crucial role in improving this disease’s prognosis.

## Background

Non-tuberculous mycobacterial pulmonary disease (NTM-PD) usually develops in middle-aged and older individuals, and is generally intractable and slowly progressive. The incidence and prevalence of NTM-PD are reportedly increasing in many parts of the world [1–7]. Increasing numbers of NTM-PD-related deaths among HIV-uninfected patients has also been reported in Japan [2] and the USA [8]. NTM-PD related deaths are expected to be a significant health problem in countries in which the population is aging.

Several population-based studies have identified the risk factors of male sex, older age, and some comorbidities for NTM-PD-related deaths [8–11]. Clinical conditions such as low body mass index (BMI) have also been shown to be associated with poor long-term prognosis of NTM-PD in some hospital-based studies [12, 13]; however, these studies had small patient cohorts. With regard to the significance of clinical deterioration of patients with NTM-PD, little information is available, particularly on in-hospital deaths. Evaluating risk factors for in-hospital mortality is crucial to improving the prognosis in patients with NTM-PD.

In this study, we used data from a nationwide database in Japan to investigate the characteristics and comorbidities of patients with NTM-PD who required unscheduled hospitalization and examined factors associated with in-hospital mortality in these patients.

## Methods

### Data source

We examined data from the Diagnosis Procedure Combination (DPC) database in Japan from July 2010 to March 2014. The database includes administrative claims data and discharge abstract data from more than 1200 hospitals, which covered 50% of the total bed capacity of acute care hospitals during the survey period. Primary diagnoses and comorbidities are recorded using International Statistical Classification of Diseases and Related Health Problems, 10th Revision (ICD-10) codes, accompanied by text data in Japanese. The database also contains the following information: age, sex, body height and weight, smoking status, grade of activity of daily living expressed as Barthel index score on admission, discharge status, therapeutic procedures, and medication use during hospitalization. This study was approved by the Institutional Review Board of The University of Tokyo and was performed in accordance with the Declaration of Helsinki. The requirement for informed consent was waived because of the anonymous nature of the data.

### Patient selection and data

We retrospectively extracted data for patients with ICD-10 code: A310 (pulmonary mycobacterial infection) and A319 (Mycobacterial infection, unspecified). We did not include patients who were diagnosed with NTM extra-pulmonary disease (ICD-10 code, A311 and A318) during the study period. We included only the last hospitalization of patients who required unscheduled hospitalization more than once during the study period. We excluded patients < 18 years of age and those with HIV infection (ICD-10 code, B20–B24). We identified comorbidities using ICD-10 codes, as shown in Table 1. Because it is difficult to distinguish between primary bronchiectasis and bronchiectasis secondary to NTM-PD [14], we did not include the comorbidity of bronchiectasis in our study.

**Table 1:** ICD-10 codes used to identify comorbidities

Comorbidity	ICD-10 codes
Pulmonary infection	J100, J110, J12–J18, J20–22, J85, J86, J690
Pulmonary aspergillosis	B44
COPD	J43, J440, J441, J449
Bronchial asthma	J45, J46
Interstitial lung disease	J841, J848, J849
Pneumothorax	J93
Congestive heart failure	I110, I500, I501, I509
Ischemic heart disease	I20–I25
Cerebrovascular disease	I60–I69
Renal disease	N00–08, N10–N19
Autoimmune disease	M05, M06, M08, M30–M35
Diabetes mellitus	E10–E14
Bone fracture	S02, S12, S22, S32, S42, S52, S62, S72, S82, S92, T02, T10, T12
Lung cancer	C33, C34
Hematological malignancy	C81–C85, C88, C90–C96
Other malignant disease	C00–C97

To evaluate weight loss, we categorized BMI in accordance with the levels of severity of anorexia nervosa in the Diagnostic and Statistical Manual of Mental Disorders, Fifth Edition (DSM-5) [15].

We used either ICD-10 codes (R042, R048, and R049) or treatment with intravenous hemostatic agents (carbazochrome sodium sulfonate and/or tranexamic acid) as indicators of clinically significant hemoptysis or bloody sputum. We then excluded patients with diagnoses of hemorrhage from organs other than lungs (ICD-10 codes shown in Table 2) and those who had undergone endoscopic procedures for hemostasis of gastrointestinal tract bleeding. We considered that arterial embolization performed in patients with hemoptysis was bronchial artery embolization.

**Table 2:** ICD-10 codes used to identify hemorrhage from organs other than lung

	ICD-10 codes
Hemorrhage from urinary system	N288, N300, N304, N309, N328, N368, N421, N421, N488, N501, E274
Hemorrhage associated with gynecological disease	N645, N830, N831, N838, N908, N921–N924, N930, N938, N939, N950, N988, O209, O441, O469, O679, O695, O720–O722, O730, O731
Hemorrhage associated with otolaryngological disease	E078, H603, H669, H738, H922, R040, R041
Intracerebral hemorrhage	I60–I62
Spinal cord hemorrhage	G951, G968
Ocular hemorrhage	H113, H168, H208, H210, H313, H350, H356, H357, H405, H431, H448, H470
Traumatic hemorrhage	S013, S051, S063, S066, S068, S098, S368, S378, T144, T794
Postoperative hemorrhage	T810, T811

In addition, we extracted data concerning oral and intravenous treatment with various drugs, including corticosteroids and anti-mycobacterial agents. We evaluated prescription of the following antibiotics: rifamycins, including rifampicin and rifabutin; ethambutol; isoniazid; macrolides, including clarithromycin and azithromycin; aminoglycosides, including streptomycin, kanamycin, and amikacin; fluoroquinolones, including levofloxacin, moxifloxacin and sitafloxacin; and imipenem/cilastatin. We then assessed the combination of rifamycin, ethambutol, and clarithromycin, which is the recommended first line therapy for *Mycobacterium avium* complex pulmonary disease (MAC-PD) in Japan [16]. We also evaluated prescription of erythromycin, which has an anti-inflammatory effect in patients with chronic airway diseases.

### Statistical analysis

We used χ^2^ tests to compare baseline characteristics, treatments, and procedures during hospitalization between patients who died during hospitalization and those who survived. We performed multivariable logistic regression to identify risk factors for all-cause in-hospital mortality. We used multiple imputation by the chained equations technique to deal with missing data on BMI, smoking history, and Barthel index scores. We included all the variables analyzed in this study in the imputation model and created 20 imputed datasets. We fitted multivariable logistic regression analyses for in-hospital mortality with generalized estimating equations to account for within-hospital clustering [17]. We obtained one set of statistical results on each imputed dataset and integrated them using Rubin’s combination rules [18]. We set statistical significance at less than 0.05 for all analyses. We performed statistical analyses using Stata/MP version 14 (StataCorp, College Station, TX, USA).

## Results

### Patient characteristics

We identified 16,280 patients with NTM-PD who required unscheduled hospitalization during the study period. We then excluded 24 patients aged < 18 years and 64 patients with HIV infection from the study, leaving 16,192 patients who were eligible for further analysis.

These patients characteristics are shown in Table 3. The median age was 78 years (interquartile range [IQR] 71–84) and 61.2% were women. The overall median BMI was 17.5 kg/m^2^ (IQR 15.4–20.0), being 18.1 kg/m^2^ (IQR 15.9–20.6) for men and 17.2 kg/m^2^ (IQR 15.2–19.5) for women. The most common comorbidity was pulmonary infection other than NTM (50.8%). As for primary diagnosis during hospitalization, pulmonary diseases accounted for 68.3% (pulmonary infection 28.8%, NTM-PD 23.1%, and other pulmonary disease 16.4%). Malignant diseases and other non-pulmonary diseases accounted for 4.1 and 27.6%, respectively.

**Table 3:** Baseline characteristics of patients with NTM pulmonary disease who required unscheduled hospitalization

	Total (%)		Death (%)		*P*-value
	*n* = 16,192		*n* = 3166		
Sex					< 0.001
Male	6283	(38.8)	1543	(48.7)	
Female	9909	(61.2)	1623	(51.3)	
Age, years					< 0.001
< 70	3400	(21.0)	455	(14.4)	
70–79	5374	(33.2)	1035	(32.7)	
≥ 80	7418	(45.8)	1676	(52.9)	
BMI, kg/m^2^					< 0.001
< 15.0	2858	(17.7)	910	(28.7)	
15.0–15.9	1627	(10.0)	371	(11.7)	
16.0–16.9	1714	(10.6)	297	(9.4)	
17.0–18.4	2533	(15.6)	365	(11.5)	
18.5–24.9	5036	(31.1)	568	(17.9)	
≥ 25.0	476	(2.9)	44	(1.4)	
Missing data	1948	(12.0)	611	(19.3)	
Activities of daily living, Barthel index					< 0.001
100	5137	(31.7)	354	(11.2)	
75–95	1553	(9.6)	159	(5.0)	
50–70	1921	(11.9)	336	(10.6)	
25–45	1225	(7.6)	296	(9.3)	
0–20	3818	(23.6)	1426	(45.0)	
Missing data	2538	(15.7)	595	(18.8)	
Smoking history					< 0.001
No	11,373	(70.2)	2083	(65.8)	
Yes	3167	(19.6)	668	(21.1)	
Missing data	1652	(10.2)	415	(13.1)	
Symptom					
Hemoptysis	2996	(18.5)	528	(16.7)	0.003
Comorbidity					
Pulmonary disease					
Pulmonary infection	8225	(50.8)	2024	(63.9)	< 0.001
Pulmonary aspergillosis	874	(5.4)	261	(8.2)	< 0.001
COPD	1527	(9.4)	362	(11.4)	< 0.001
Bronchial asthma	1158	(7.2)	181	(5.7)	< 0.001
Interstitial lung disease	1171	(7.2)	384	(12.1)	< 0.001
Pneumothorax	730	(4.5)	183	(5.8)	< 0.001
Non-pulmonary disease					
Congestive heart failure	2279	(14.1)	650	(20.5)	< 0.001
Ischemic heart disease	1123	(6.9)	184	(5.8)	0.006
Cerebrovascular disease	1248	(7.7)	243	(7.7)	0.94
Renal disease	853	(5.3)	218	(6.9)	< 0.001
Autoimmune disease	1225	(7.6)	198	(6.3)	0.002
Diabetes mellitus	2289	(14.1)	460	(14.5)	0.48
Bone fracture	831	(5.1)	114	(3.6)	< 0.001
Malignant disease					
Lung cancer	532	(3.3)	176	(5.6)	< 0.001
Hematological malignancy	234	(1.4)	71	(2.2)	< 0.001
Other malignant disease	1058	(6.5)	231	(7.3)	0.053

### Treatments during hospitalization

Treatments during hospitalization are shown in Table 4; 44.6% of the 16,192 patients had not received any antibiotics with antimicrobial activity against NTM during the hospitalization, 3.2% had received monotherapy with erythromycin, and 15.2% had been treated with combination therapy including rifamycin, ethambutol and clarithromycin. Corticosteroids were prescribed for 20.9% of all patients.

**Table 4:** Treatment of study patients during hospitalization

	Total (%)		Death (%)		*P-*value
	*n =* 16,192		*n =* 3166		
Medications prescribed during hospitalization					
No use of NTM drugs^a^	7215	(44.6)	1504	(47.5)	< 0.001
Erythromycin only	526	(3.2)	61	(1.9)	< 0.001
RIF, EMB, CLR	1676	(10.4)	232	(7.3)	< 0.001
RIF, EMB, CLR, +FQ and/or AG	778	(4.8)	188	(5.9)	0.001
Corticosteroids^b^	3383	(20.9)	1069	(33.8)	< 0.001
Treatment procedure					
Bronchial artery embolization	250	(1.5)	22	(0.7)	< 0.001
Mechanical ventilation	1518	(9.4)	957	(30.2)	< 0.001

^a^NTM drugs include the followings: rifampicin and rifabutin; ethambutol; isoniazid; macrolides, including clarithromycin and azithromycin; aminoglycosides, including streptomycin, kanamycin and amikacin; fluoroquinolones, including levofloxacin, moxifloxacin and sitafloxacin; and imipenem/cilastatin

^b^Corticosteroids include those administered both orally and intravenously

*Abbreviations*: *AG* aminoglycoside, *CLR* clarithromycin, *EMB* ethambutol, *FQ* fluoroquinolone, *NTM* non-tuberculous mycobacterial pulmonary disease, *RIF* rifampicin

### All-cause in-hospital mortality

Overall, 3166 patients (19.6%) died during their unscheduled hospitalizations (Table 3). The median age of these patients was 80 years (IQR 74–85). The median BMI of all patients who died in hospital was 16.0 kg/m^2^ (IQR 14.2–18.4), comprising 16.6 kg/m^2^ (IQR 14.8–19.1) for men and 15.4 kg/m^2^ (IQR 13.7–17.6) for women. The median length of hospital stay was 18 days (IQR 10–34) in all patients and 21 days (IQR 8–44) in patients who died during hospitalization. Two-thirds of the patients who required mechanical ventilation died during the hospitalization (Table 4).

Table 5 shows the results of the multivariable logistic regression analysis for all-cause in-hospital mortality. Higher in-hospital mortality was associated with male sex, lower BMI, lower Barthel index score, hemoptysis, and comorbidities, including pulmonary infection other than NTM, pulmonary aspergillosis, interstitial lung disease, pneumothorax, congestive heart failure, renal disease, and malignant disease.

**Table 5:** Results of multivariable logistic regression analysis with multiple imputation for all-cause in-hospital mortality in patients with NTM pulmonary disease who required unscheduled hospitalization

	OR	95% CI	*P*-value
Sex			
Male	1.89	(1.70–2.10)	< 0.001
Age, years			
< 70	reference		
70–79	1.12	(0.98–1.29)	0.11
≥ 80	1.04	(0.90–1.20)	0.56
BMI, kg/m^2^			
< 15.0	3.15	(2.74–3.62)	< 0.001
15.0–15.9	2.15	(1.81–2.54)	< 0.001
16.0–16.9	1.57	(1.31–1.87)	< 0.001
17.0–18.4	1.31	(1.13–1.52)	< 0.001
18.5–24.9	reference		
≥ 25.0	0.83	(0.58–1.18)	0.30
Activities of daily living, Barthel index			
100	reference		
75–95	1.45	(1.19–1.78)	< 0.001
50–70	2.58	(2.18–3.06)	< 0.001
25–45	3.74	(3.07–4.55)	< 0.001
0–20	7.72	(6.68–8.92)	< 0.001
Smoking history			
Yes	0.89	(0.78–1.01)	0.077
Symptom			
Hemoptysis	1.21	(1.07–1.37)	0.002
Comorbidity			
Pulmonary disease			
Pulmonary infection	1.70	(1.54–1.87)	< 0.001
Pulmonary aspergillosis	1.65	(1.39–1.96)	< 0.001
COPD	0.96	(0.83–1.12)	0.63
Bronchial asthma	0.81	(0.68–0.98)	0.027
Interstitial lung disease	2.77	(2.37–3.24)	< 0.001
Pneumothorax	1.40	(1.14–1.73)	0.001
Non-pulmonary disease			
Congestive heart failure	1.62	(1.43–1.83)	< 0.001
Ischemic heart disease	0.79	(0.66–0.95)	0.011
Cerebrovascular disease	0.75	(0.63–0.88)	0.001
Renal disease	1.46	(1.21–1.76)	< 0.001
Autoimmune disease	0.85	(0.72–1.01)	0.072
Diabetes mellitus	1.03	(0.90–1.18)	0.65
Bone fracture	0.49	(0.39–0.61)	< 0.001
Malignant disease	2.07	(1.79–2.39)	< 0.001

## Discussion

In this study, we analyzed in-hospital mortality using data of more than 16,000 patients with NTM-PD drawn from a nationwide database in Japan. Pulmonary diseases accounted for 68.3% of primary diagnoses during these patients’ hospitalizations. The results of multivariable logistic regression analysis showed that male sex, lower BMI, lower Barthel index score, and hemoptysis were associated with higher in-hospital mortality. Several comorbidities were also associated with higher mortality.

Several previous population-based studies have evaluated risk factors for NTM-related deaths. These studies identified older age [8, 9, 11, 19] and male sex [9–11, 19] as potential risk factors for NTM-related deaths. Several comorbid diseases were also shown to be possible risk factors, including chronic obstructive pulmonary disease (COPD) [8, 11], lung cancer [10, 11, 19], bronchial asthma [10], pneumonia [10], and interstitial lung disease [11]. Bloody sputum was also associated with mortality in one single center study [20].

In the present study, we found that most of the participants (50.8%) had comorbid pulmonary infections in addition to NTM. Furthermore, pulmonary infection was significantly associated with in-hospital mortality, whereas, COPD and bronchial asthma were not. It remains unknown why COPD and bronchial asthma were not associated with higher in-hospital mortality. One possibility is that some of the patients hospitalized for exacerbations of COPD or bronchial asthma had better treatment responses.

Almost 90% of NTM-PD in Japan is reportedly MAC-PD [3]. In the present study, 15.2% of the patients received combination therapy including rifampicin, ethambutol, and clarithromycin, which is a standard regimen for MAC-PD, whereas 44.6% did not receive any antibiotics that target NTM. It seems likely that a relatively large proportion of patients in our study required unscheduled hospitalization for management of comorbid diseases or conditions.

In a previous study of 178 patients with NTM-PD from Oregon, USA, regular use of immunosuppressive medication was a risk factor for death [19]. In our study, about one third of patients who received corticosteroids after admission died during hospitalization. It is possible that most of the patients who were treated with corticosteroids had severe comorbidities on admission. Further studies are needed to elucidate the association between regular use of corticosteroids and prognosis of NTM-PD.

This study included BMI data in the multivariable analysis for mortality; to the best of our knowledge, no published studies have examined BMI prior to death in patients with NTM-PD. However, several studies have reported an association between weight loss and development of NTM-PD [12, 13, 21–23]. In this study, patients with NTM-PD who required unscheduled hospitalization had remarkably low BMIs, the median being 17.5 kg/m^2^. Furthermore, lower BMI was strongly associated with higher in-hospital mortality. Our findings are in line with those of other hospital-based studies assessing long-term prognosis of MAC-PD patients in Japan, which have repeatedly shown that a BMI of less than 18.5 kg/m^2^ is associated with higher mortality [12, 13]. It is possible that most of the patients who died during their hospitalizations had cachexia, which is recognized as a complex metabolic syndrome [24].

Development of more effective anti-mycobacterial drugs may be crucial to preventing progression of NTM-PD. However, such drugs may not completely prevent progression of NTM-PD because some patients are likely to have polyclonal and mixed NTM infections acquired from the environment, as well as reinfection with NTM after treatment [25, 26]. Taken together, exploring host factors (such as the mechanisms by which severe weight loss affects susceptibility to, and progression of, NTM-PD) may be important in improving the prognosis of this disease. In fact, two previous studies have reported a possible role for inappropriately secreted adipokines in the pathogenesis of NTM-PD [27, 28]; however, their results were inconsistent and thus require further investigation.

Several limitations must be acknowledged. First, mild cases of NTM-PD without respiratory complications may have not been recorded by the attending physician; this would have resulted in low sensitivity for the diagnosis of mild cases of NTM-PD. NTM-PD is more likely to be diagnosed when it is has resulted in moderate to severe respiratory symptoms. Second, we may have underestimated the proportion of patients who were receiving combination therapy for MAC-PD because anti-mycobacterial drugs prescribed in the outpatient settings are usually withdrawn on admission in more severe cases. This would have prevented us from accurately evaluating the association between antibacterial therapies for NTM-PD and in-hospital mortality.

## Conclusions

In the present study of data drawn from a nationwide inpatient database in Japan, we identified multiple factors associated with in-hospital mortality of NTM-PD. In particular, we found associations between pulmonary infection other than NTM and lower BMI and higher in-hospital mortality. Controlling for severe weight loss and comorbidities may play a key role in improving the prognosis of this disease.

## Data Availability

Data cannot be made publicly available for ethical reasons as the data are patient data. The data are available to interested researchers upon reasonable request to the corresponding author, pending ethical approval.

## Abbreviations

BMI: Body mass index
COPD: Chronic obstructive pulmonary disease
DPC: Diagnosis Procedure Combination
DSM-5: Diagnostic and Statistical Manual of Mental Disorders, Fifth Edition
ICD-10: International Statistical Classification of Diseases and Related Health Problems, 10th Revision
IQR: Interquartile range
MAC-PD: *Mycobacterium avium* complex pulmonary disease
NTM-PD: Non-tuberculous mycobacterial pulmonary disease
